# Dual Hormonal Presentation in a Rare Sellar Gangliocytoma: Diagnostic and Pathological Insights from a Collision Tumor

**DOI:** 10.1055/a-2753-9561

**Published:** 2025-12-03

**Authors:** Oyku Ozturk, Mehmet A. Inan, Muhittin Akalin, Muammer M. Sahin, Emrah Celtikci

**Affiliations:** 1 Department of Neurosurgery, Gazi University Faculty of Medicine, Ankara, Türkiye; 2 Department of Pathology, Gazi University Faculty of Medicine, Ankara, Türkiye; 3 Department of Otorhinolaryngology/Head and Neck Surgery, Gazi University Faculty of Medicine, Ankara, Türkiye

**Keywords:** acromegaly, collision tumor, cushing disease, gangliocytoma, pituitary adenoma

## Abstract

**Background:**

Gangliocytomas of the sellar region are rare, well-differentiated, benign tumors that may coexist with pituitary adenomas, forming so-called “collision tumors.” These lesions often present with endocrine dysfunction, most commonly acromegaly.

**Case Description:**

We report a 69-year-old female who presented with drug-resistant headaches, acromegalic features, and signs of Cushing's disease. Magnetic resonance imaging showed a sellar mass with extension into the clivus. Endoscopic transsphenoidal resection revealed a tumor composed of ganglion cells and adenohypophyseal components. Immunohistochemistry confirmed a diagnosis of gangliocytoma coexisting with a hormone-secreting pituitary adenoma. Postoperative hormonal normalization was achieved.

**Conclusion:**

Sellar gangliocytomas with dual hormonal activity pose diagnostic challenges and highlight the importance of histological and immunohistochemical evaluation. Awareness of these rare entities can prevent misdiagnosis and support appropriate surgical management.

## Introduction

Pituitary adenomas represent the most common tumors of the sellar region, accounting for 85 to 90% of sellar lesions and 10 to 15% of all intracranial neoplasms. 1 In contrast, gangliocytomas are exceptionally rare, well-differentiated, and slow-growing benign tumors arising from the posterior pituitary region. These lesions comprise less than 1% of all sellar tumors. They are now classified under the glioneuronal and neuronal tumors category in the 2021 World Health Organization classification of central nervous system tumors.

Sellar gangliocytomas may occur in isolation or coexist with pituitary adenomas, most frequently with growth hormone (GH)-secreting subtypes. The majority of mixed lesions—often referred to as collision tumors—present with acromegaly due to GH excess, followed by hyperprolactinemia and Cushing's disease. 2 3 4 5

Since the first description of an intrasellar gangliocytoma by Greenfield in 1919, approximately 207 cases have been reported in the literature. 4 Herein, we present a rare case of a sellar gangliocytoma manifesting with both acromegaly and Cushing's syndrome, managed via endoscopic endonasal resection. This report aims to contribute to the limited existing literature by discussing the clinicopathological features, differential diagnosis, and potential histogenetic mechanisms of these uncommon neoplasms.

## Case Report

**Fig. 1:**
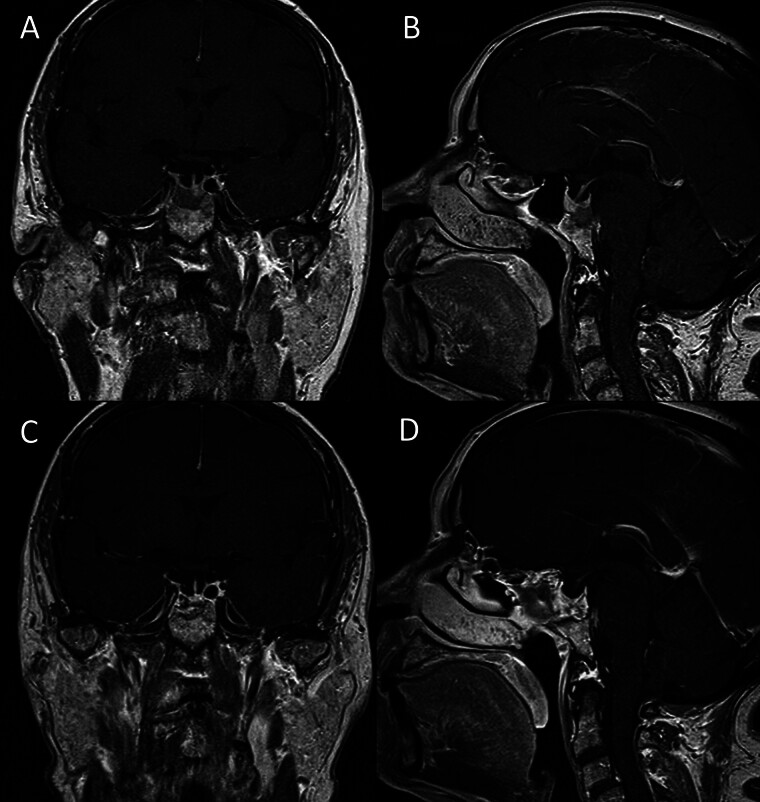
Preoperative coronal ( **A** ), sagittal ( **B** ), and postoperative coronal ( **C** ), sagittal ( **D** ) T1-weighted MRI scans showing a hypointense, hypovascular sellar lesion with clival extension.

A 69-year-old woman presented with a 5-year history of medication-resistant headache, accompanied by features of acromegaly and a Cushingoid appearance. Laboratory investigations revealed elevated levels of adrenocorticotropic hormone (ACTH) at 133.9 pg/mL (ULN: 63.3 pg/mL) and insulin-like growth factor 1 (IGF-1) at 190.3 ng/mL (ULN: 163 ng/mL). The 8 a.m. cortisol level was 20.59 µg/dL ( *n* : 5.27–22.45 µg/dL), the overnight salivary cortisol was 10.7 µg/dL (<0.145–0.25 µg/dL), and the 24-hour urine free cortisol was 179 µg/24 hours (36–137 µg/24 hours). Low-dose dexamethasone suppression tests of 1 mg and 2 mg resulted in 7.58 µg/dL ( *n* < 1.8 µg/dL) and 6.82 µg/dL (<1.8 µg/dL), respectively. An 8 mg high-dose dexamethasone suppression test, applied to confirm the diagnosis, resulted in 1.25 µg/dL, indicating pituitary-originated Cushing's disease with > 50% suppression of basal cortisol. The endocrinology team concluded that inferior petrosal sinus sampling was not required. Moreover, magnetic resonance imaging (MRI) clearly showed a hypointense, hypovascular mass originating from the right side of the pituitary gland and extending into the clivus ( Fig. 1A, B ). The lesion appeared distinct from normal pituitary tissue.

**Fig. 2:**
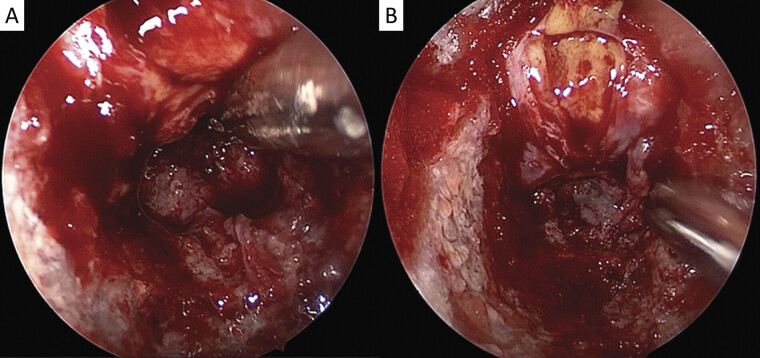
Intraoperative image of an encapsulated off-white lesion eroding the sphenoid bone ( **A, B** ).

The patient underwent endoscopic endonasal transsphenoidal resection. Intraoperatively, the tumor appeared as an off-white, encapsulated mass with medium firmness, causing localized erosion of the sphenoid bone ( Fig. 2 ). Gross-total resection was achieved, as confirmed by postoperative MRI ( Fig. 1C, D ).

**Fig. 3:**
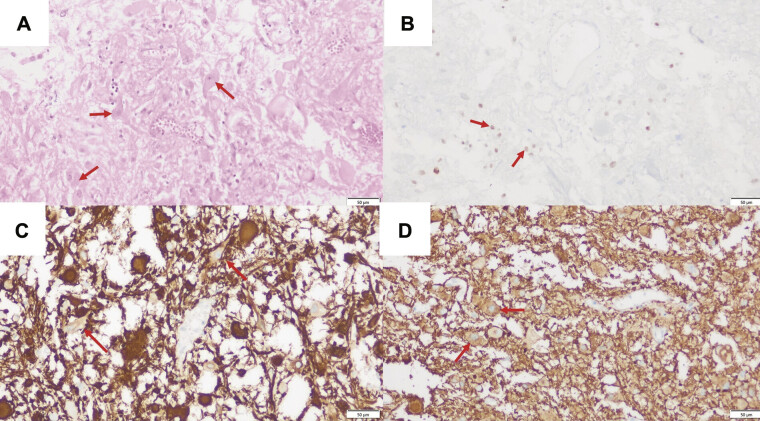
Hematoxylin and eosin stain of sellar gangliocytoma showing multipolar ganglion cells that contained prominent nucleoli and Nissl bodies ( **A** , ×200; red arrows indicate ganglion cells). Ganglion cells typically stained positive for neurofilament ( **C** , ×200; red arrows indicate neurofilament-positive cell bodies and processes) and synaptophysin ( **D** , ×200; red arrows), while round mononuclear adenohypophyseal cells stained with T-pit. ( **B** , ×200; red arrows).

Histopathologic examination revealed a biphasic lesion composed of large, multipolar ganglion cells with prominent nucleoli and Nissl bodies, consistent with gangliocytoma ( Fig. 3A ). These cells stained positively for synaptophysin and neurofilament protein (NFP; Fig. 3C, D ), while monomorphic adenohypophyseal cells stained with T-pit, indicating coexisting corticotroph adenoma components ( Fig. 3B ).

The postoperative course was uneventful. Hormonal follow-up showed progressive normalization with corticosteroid support. At the final follow-up, ACTH and IGF-1 levels had decreased to 36.6 pg/mL and 120.7 ng/mL, respectively, with complete resolution of Cushingoid and acromegalic features.

## Discussion

Sellar gangliocytomas are exceedingly rare, benign neoplasms of neuronal origin, accounting for less than 1% of sellar region tumors and less than 2% of all intracranial neoplasms. These tumors may occur in isolation or, more commonly, in conjunction with pituitary adenomas, forming collision tumors. Among these, GH-secreting adenomas are the most frequent coexisting subtype, often manifesting clinically as acromegaly, followed by prolactinomas and corticotroph adenomas causing Cushing's disease. 2 3 4 5

The clinical presentation varies depending on the hormonal activity of the adenomatous component, although nonfunctioning gangliocytomas can still cause mass effect, leading to headaches or visual disturbances. In the present case, the coexistence of both acromegaly and Cushingoid features reflected hormonal activity consistent with GH and ACTH excess.

Several hypotheses have been proposed regarding the histogenesis of sellar gangliocytoma–pituitary adenoma collision tumors:

- Hypothalamic hormone hypothesis: ectopic hypothalamic hormone production (e.g., GHRH or CRH) by ganglion cells stimulates pituitary cell proliferation.
- Transdifferentiation theory: suggests that neuronal differentiation of preexisting adenoma cells is mediated via nerve growth factor (NGF) and its receptor NGFR, which have been detected in some adenoma subtypes. Co-expression of Neurofilament proteins (NFP) in both tumor components supports this view. 1
- Common progenitor cell theory: both neuronal and adenohypophyseal elements may arise from a shared multipotent stem cell. 6
- Simultaneous stimulation hypothesis: a shared mitogenic event may trigger parallel proliferation of both cell types. 7

While transdifferentiation currently remains the most widely accepted theory, the precise pathogenesis is yet to be elucidated.

Surgical resection remains the mainstay of treatment. In a review of reported cases, 74% were managed with surgery alone, while others received adjuvant therapy including radiotherapy or pharmacologic treatment. 4 In most instances, gross-total resection is achievable, and the prognosis is excellent. Importantly, the presence of ganglionic differentiation does not appear to alter recurrence risk or long-term outcomes. 3 5

In our case, the lesion was successfully resected via an endoscopic endonasal approach, with postoperative normalization of hormone levels. No complications or recurrence were observed during follow-up, aligning with existing literature that emphasizes the favorable prognosis of these tumors when complete resection is achieved.

## Conclusion

Sellar gangliocytomas are exceedingly rare, benign neuronal tumors that may present either in isolation or as part of a collision tumor with pituitary adenomas. Diagnosis based solely on clinical and radiological findings remains challenging due to overlapping features with more common pituitary neoplasms. Histopathologic analysis following surgical resection is essential for definitive diagnosis.

Among the proposed mechanisms of tumorigenesis, neural transdifferentiation of pituitary adenoma cells is the most widely accepted, supported by immunohistochemical and molecular findings in mixed tumors. Importantly, the presence of a ganglionic component does not appear to impact prognosis, recurrence risk, or therapeutic strategy.

In this case, complete endoscopic resection resulted in both radiologic and hormonal remission without complications. This case reinforces the importance of considering mixed sellar pathologies in hormonally active pituitary tumors and contributes to the limited existing data on this rare entity.
